# Comparing the efficacy of electronic cognitive behavioral therapy to medication and combination therapy for generalized anxiety disorder: a quasi-experimental clinical trial

**DOI:** 10.3389/fpsyt.2023.1194955

**Published:** 2023-12-06

**Authors:** Callum Stephenson, Elnaz Moghimi, Yijia Shao, Anchan Kumar, Caitlin S. Yee, Shadé Miller, Anthi Stefatos, Maedeh Gholamzadehmir, Zara Abbaspour, Jasleen Jagayat, Amirhossein Shirazi, Tessa Gizzarelli, Gilmar Gutierrez, Ferwa Khan, Charmy Patel, Archana Patel, Megan Yang, Mohsen Omrani, Nazanin Alavi

**Affiliations:** ^1^Department of Psychiatry, Faculty of Health Sciences, Queen’s University, Kingston, ON, Canada; ^2^Department of Psychology, Faculty of Arts and Science, University of Toronto, Toronto, ON, Canada; ^3^Centre for Neuroscience Studies, Faculty of Health Sciences, Queen’s University, Kingston, ON, Canada; ^4^OPTT Inc., Digital Media Zone, Ryerson University, Toronto, ON, Canada

**Keywords:** mental health, anxiety, generalized anxiety disorder, cognitive behavioral therapy, psychotherapy, electronic cognitive behavioral therapy, mental health care

## Abstract

**Background:**

Generalized anxiety disorder (GAD) is a debilitating mental health disorder with first-line treatments include cognitive behavioral therapy (CBT) and pharmacotherapy. CBT is costly, time-consuming, and inaccessible. Electronic delivery (e-CBT) is a promising solution to address these barriers. However, due to the novelty of this intervention, more research testing the e-CBT efficacy independently and in conjunction with other treatments is needed.

**Objective:**

This study investigated the efficacy of e-CBT compared to and in conjunction with pharmacotherapy for GAD.

**Methods:**

This study employed a quasi-experimental design where patients selected their preferred treatment modality. Patients with GAD were enrolled in either e-CBT, medication, or combination arms. The 12-week e-CBT program was delivered through a digital platform. The medications followed clinical guidelines. The efficacy of each arm was evaluated using questionnaires measuring depression, anxiety, and stress severity, as well as quality of life.

**Results:**

There were no significant differences between arms (N e-CBT = 41; N Medication = 41; N Combination = 33) in the number of weeks completed or baseline scores. All arms showed improvements in anxiety scores after treatment. The medication and combination arms improved depression scores. The e-CBT and Combination arms improved quality of life, and the combination arm improved stress scores. There were no differences between the groups in depression, anxiety, or stress scores post-treatment. However, the combination arm had a significantly larger improvement in quality of life. Gender and treatment arm were not predictors of dropout, whereas younger age was.

**Conclusion:**

Incorporating e-CBT on its own or in combination with pharmaceutical interventions is a viable option for treating GAD. Treating GAD with e-CBT or medication appears to offer significant improvements in symptoms, with no meaningful difference between the two. Combining the treatments also offer significant improvements, while not necessarily superior to either independently. The findings suggest that all options are viable. Taking the patient’s preferred treatment route based on their lifestyle, personality, and beliefs into account when deciding on treatment should be a priority for care providers.

## 1 Introduction

An estimated 970 million people suffer from mental health disorders globally (1). Anxiety disorders are the most common mental health disorder, having an estimated lifetime prevalence of 30%, with generalized anxiety disorder (GAD) being the most prevalent anxiety disorder (1). Despite the high prevalence, less than half of people with mental health disorders can receive treatment (2). To meet the large demand for treatment, the mental health care system requires accessible, cost-effective, and scalable solutions.

Currently, the treatments of choice for GAD are psychotherapy and pharmacotherapy, with both routes showing significant improvements in anxiety symptoms compared to control (3). Regarding psychotherapy, 12–20 sessions of individual cognitive behavioral therapy (CBT) is considered the first-line treatment (3–5). CBT teaches the patient useful skills and strategies to address their anxieties while aiming to reshape their cognitions and behaviors (3, 5). Individual CBT can improve patient quality of life, decrease psychological distress, and provide long-term benefits (6–10). The recommended first-line pharmacotherapies for treating GAD are serotonin-targeting drugs, involved in the body’s stress responses (3, 4), specifically selective serotonin reuptake inhibitors (SSRIs) and serotonin-norepinephrine reuptake inhibitors (SNRIs). SSRIs increase the level of serotonin in the brain by blocking neuron receptors from reabsorbing serotonin. SNRIs work very similarly, blocking the reuptake of serotonin, with the addition of blocking norepinephrine reabsorbtion (3, 4). SSRIs and SNRIs are first-line therapies for treating GAD in part for their efficacy, but also the relatively low risk of adverse effects compared to other pharmaceutical interventions (4, 11). SSRIs and SNRIs have shown comparable efficacy in treating anxiety disorders (3, 4, 12–16). Regarding the combination of in-person CBT and pharmacotherapy, there is conflicting evidence as to whether the efficacy is augmented (17–22). Due to this, current guidelines do not support the routine combination of CBT and pharmacotherapy (3). However, if a patient experiences minimal improvement from one alone, then combining may have beneficial effects (3).

While pharmacotherapy and CBT are effective, neither is without drawbacks. Stigma, potential adverse effects, polypharmacy, and personal reasons associated with pharmacotherapy may deter many patients from receiving this treatment (3). With CBT, high costs, long wait lists, privacy concerns, and inflexible treatment times often make the therapy inaccessible (23). Moreso, some patients may feel that they only wish to receive medication, as they feel psychotherapy will not be a good fit for them. Depending on the personal factors of each patient, deciding on which treatment route to go down can be difficult. Some patients may be averse to using medications, while some may be less psychologically minded and wish to not participate in psychotherapy. Additionally, some patients may be intrigued by the option of a combination approach. Incorporating patient preference into the intervention decision can increase adherence and treatment outcomes (24–27). With psychotherapy and pharmacotherapy having generally the same efficacy (17, 28), developing more accessible interventions that fit the individual needs and wishes of each patient without sacrificing the quality of care is paramount.

One way to address some accessibility concerns of CBT is to deliver it through the internet (e-CBT). In this paper, “e-CBT” refers to any form of electronically-delivered CBT program. e-CBT has demonstrated clinical efficacy, can increase treatment adherence and satisfaction, and offers comparable results to in-person CBT (29–33). Given the structured nature of CBT, pre-designed content can be provided to patients, allowing them to access it anywhere at any time, saving healthcare providers time and costs, while increasing care capacity (33). Many studies show e-CBT to be effective in treating anxiety symptoms, with differing levels of effectiveness across delivery modalities, with digital self-help interventions being extremely popular (29–33). While self-help programs do offer benefits, the incorporation of therapist engagement can further increase effectiveness (33–35). One form of e-CBT delivery is *live* psychotherapy (synchronous delivery through video calls with the patient). While this modality offers geographical accessibility improvements (patients do not have to travel to their appointments), the time commitment for a care provider remains identical to the in-person form. Therefore, asynchronous delivery of e-CBT with therapist supervision is a promising compromise to achieve both accessibility and scalability benefits. Although e-CBT offers comparable results to in-person CBT when treating GAD, e-CBT has yet to be compared directly to the efficacy of pharmacotherapy, and a combination of the two. Offering the option of e-CBT through a secure and accessible virtual platform that could have comparable effects to medication could be a promising solution to many accessibility concerns associated with in-person CBT. Moreover, investigating whether combining e-CBT and medication has different effects from in-person CBT and medication can inform future clinical guidelines.

## 2 Objectives and hypothesis

In this study, a secure and scalable e-CBT program was delivered to individuals with GAD. This program was offered through the Online Psychotherapy Tool (OPTT), a secure cloud-based platform designed specifically for the online delivery of psychotherapy (33, 36–39). Twelve weekly e-CBT modules mirrored in-person, individual CBT content. Patients were offered either e-CBT, medication, or a combination of e-CBT and medication. The following project aimed to investigate the efficacy of e-CBT in the treatment of GAD compared to and in conjunction with current pharmacotherapy strategies. Using OPTT, it was hypothesized that this psychotherapy intervention would improve patient quality of life and decrease symptom severity in individuals with GAD, independent of medication. The primary outcome measurement was a change in GAD-7 score.

## 3 Methodology and design

### 3.1 Design

This quasi-experimental study enabled patients to choose which treatment they preferred to receive. This research design aimed to be naturalistic by mimicking the decisions made by patients and physicians regarding their autonomy to choose a course of treatment. As in a non-research clinical setting, following the patient assessment, the physician treating the patient offered their recommended course of treatment but allowed the patient to ultimately decide on which intervention they would receive. This design allowed the patient to make an educated decision on their care in an autonomous setting where they had the treatment arms explained in detail to them by the letter of information along with an expert (physician) offering their recommended course of treatment. If a participant had no preference, they would still be asked if they were content proceeding with the physician’s recommendation. The treatments provided within the study also aimed to replicate evidence-based best practice clinical guidelines for the treatment of GAD. All procedures were approved by the Queen’s University Health Sciences and Affiliated Teaching Hospitals Research Ethics Board (HSREB). This study was registered at ClinicalTrials.gov (NCT04478526).

### 3.2 Participants

Participants (*N* = 115; e-CBT *N* = 41; Medication *N* = 41; Combination *N* = 33) were recruited at Queen’s University from outpatient psychiatry clinics at Kingston Health Sciences Centre sites (Hotel Dieu Hospital and Kingston General Hospital), Providence Care Hospital, family doctors, physicians, clinicians, and self-referrals in Kingston, Ontario, Canada. Interested participants met with a research coordinator who provided them with a study letter of information that they read before providing written informed consent. Additionally, it was explained to the participants that they would not always have access to their care provider and that the program was not to be used as a crisis resource. Once informed consent was provided, a psychiatrist on the research team evaluated the patients through secure video appointments. During these appointments, a diagnosis of GAD was confirmed using the Diagnostic and Statistical Manual of Mental Disorders, 5th Edition (DSM-5) (40). Inclusion criteria included the age of 18 or over at the start of the study, having a diagnosis of GAD according to the DSM-5, having the competence to consent to participate, having the ability to speak and read English, and having consistent and reliable access to the internet. Exclusion criteria included active psychosis, acute mania, severe alcohol, or substance use disorder, and active suicidal or homicidal ideation. Participants were excluded if they were currently receiving another form of psychotherapy as this could have confounding effects on the efficacy of treatment. If a participant was already on an antidepressant medication, it was required that their medication remain unchanged for at least 6 weeks before the start of the study, and during the study. Any participants that were excluded were connected with appropriate resources and still offered standard treatments outside of the research environment (i.e., medication, therapy, etc.).

### 3.3 Procedures

At baseline, patients completed a demographic questionnaire and the following clinically validated symptomatology questionnaires: The Quality-of-Life Enjoyment and Satisfaction Questionnaire – Short Form (Q-LES-Q-SF) (41), The Generalized Anxiety Disorder – 7 Item Questionnaire (GAD-7) (42), and The 42-Item Depression Anxiety Stress Scale (DASS-42) (43). The GAD-7 and DAS-42 allowed for a comprehensive assessment of anxiety, stress, and depressive symptoms while the Q-LES-Q allowed observation of changes in quality of life. All patients completed the questionnaires at baseline, (T0), mid-point (week 6; T1), and post-treatment (week 12; T2). Patients in the e-CBT or combination arms completed the questionnaires directly through OPTT. Patients in the medication arm completed the questionnaires during their appointments. If deemed eligible for the study, patients were presented with all three arms of the study by the psychiatrist who discussed the recommended treatment plan (3). In collaboration with the psychiatrist and based on what the patient felt fit their personal needs best, the participant decided which treatment arm they would be in. Participation in the study was discontinued if a participant was non-compliant with their treatment program. Pharmacotherapy noncompliance was defined as stopping altogether or skipping two or more days in a row regarding medication, this was considered stopping the medication altogether or skipping more than 3 days of doses in a row. With e-CBT treatment, non-compliance was defined as missing more than two consecutive weeks of e-CBT sessions without contact. Patients were not sent their next session and assignments on OPTT until they submitted their content from the previous session by their predetermined due date. If a patient was deemed to be in an acute crisis by self-report or by the psychiatrist in charge of their care, their treatment was halted and they were directed to the proper resources (e.g., emergency department, crisis lines, etc.).

### 3.4 e-CBT intervention

All e-CBT modules were designed to mirror standard in-person CBT for GAD (3, 5, 33). The therapy emphasized the interconnectivity of thoughts, behaviors, emotions, physical reactions, and environment (5, 33). To achieve this, the program presented relevant information, taught coping skills, and encouraged patients to practice these skills through homework assignments. Coping skills included actions such as deep breathing and meditation, goal setting, thought recording, and activity scheduling (5). Through the course of this program, patients were taught how to refocus their beliefs and thoughts to more realistic states where they could better cope with their anxiety.

Patients completed approximately 30 e-CBT slides each week through the OPTT platform. Each session was expected to last approximately 50 min. The slides highlighted a different topic each week and included general information, an overview of skills, and homework on that topic. The homework was submitted through OPTT and reviewed by a care provider assigned to the patient. Care providers gave personalized feedback every week to their patients within 3 days of submission. Patients had access to these online sessions at any point throughout the week and could complete them in multiple blocks or all at once before their due date. Weekly homework submissions for feedback were mandatory before being eligible for the next session. Feedback was reviewed by a psychiatrist on the team before submission. A description of each e-CBT session can be found below (Table 1).

**Table 1:** Overview of each e-CBT session.

Session	Description
*1 – What is Generalized Anxiety Disorder?*	Provides expectations for the course and introduces anxiety and CBT.
*2 – The 5-Part Model*	Introduces the concept of the 5-Part Model and how a situation, thoughts, feelings, physical reactions, and behaviors are connected and how they interact.
*3 – Strategies for Stressful Situations*	Provides an overview of helpful strategies that can be used in stressful situations including pleasurable activities and helpful breathing techniques.
*4 – Situation, Thoughts, Feelings, Physical Reactions, & Behaviors*	Provides a further detailed exploration of the 5 Part Model and how changes in one area can affect the other 4 parts.
*5 – The Thought Record*	Highlights the first three columns of the Thought Record; a tool used to help understand the connection between feelings, behaviors, and thoughts. The first three columns include the situation, followed by the feelings and automatic thoughts associated with it.
*6 – Automatic Thoughts*	This delves into the role of automatic thoughts and how they influence feelings. The focus of this session is to understand how to identify automatic thoughts and specifically identify the most dominant idea, or “hot thought” when presented with a stressful situation. Common thinking errors are also discussed in this session.
*7 – Activity Scheduling*	Provides a break from learning about the Thought Record and instead explains how to use an Activity Record; a tool designed to record and plan weekly activities. This session focuses on how tracking activities can inform mood changes and reinforce the scheduling of pleasurable activities.
*8 – Evidence*	Focuses on the fourth and fifth columns of the Thought Record, which is designed to help gather the information that supports or does not support the identified hot thought.
*9 – Alternative & Balanced Thinking*	Focuses on the final two columns of the Thought Record which reflects on the evidence columns to help find an alternative or balanced view of the situation. The last column invites the viewer to re-rate their feelings based on the completion of the Thought Record.
*10 – Experiments*	Explain the importance of conducting experiments to start believing alternative or balanced thoughts from the Thought Record and initiating changes in ineffective thinking patterns.
*11 – Action Plans*	Centered around identifying a problem that needs to be solved and provides a framework for creating a plan for solving the problem.
*12 – Review*	The final session is a review of the course and summarizes the main CBT concepts and tools that have been taught throughout the program.

#### 3.4.1 Care providers

All care providers on the research team had experience in psychotherapy delivery and were trained by the lead psychiatrist who is an expert in online psychotherapy delivery. During training, care providers also went through each module and learned the specific concepts and skills covered during the program. Before working with any patients, care providers were given practice feedback on simulated sessions which were assessed by a psychotherapist on the team to ensure that the quality of care was adequate. Moreover, homework feedback was only sent to the patient after it was read, edited, and approved by the lead therapist involved in their care. Any issues regarding OPTT were handled through OPTT technical support which could be accessed at any time.

### 3.5 Pharmacotherapy intervention

**Figure 1:**
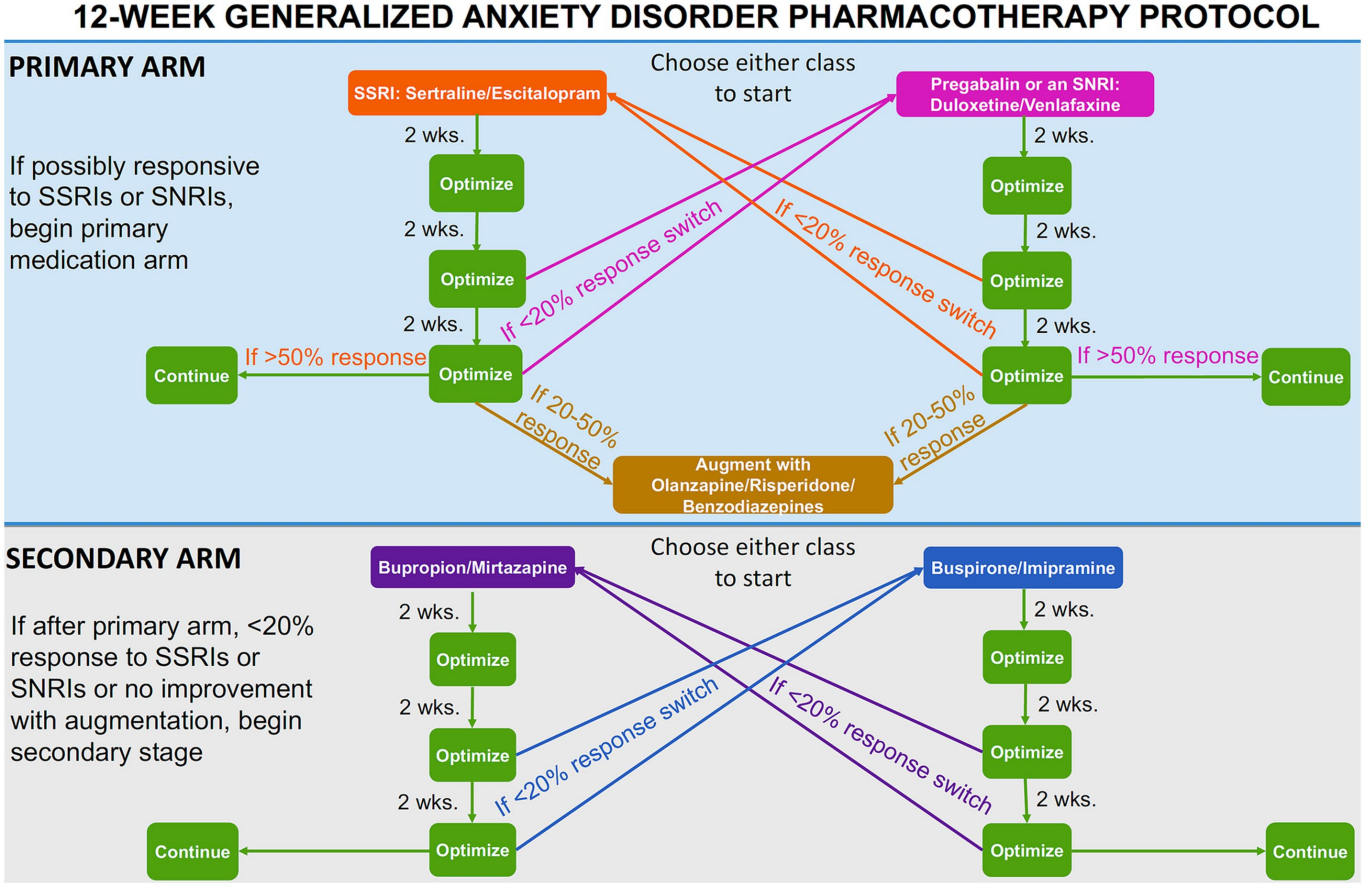
Pharmacotherapy protocol flow-chart.

In this arm, the psychiatrists suggested medications according to Canada’s best practice guidelines for the treatment of GAD^3^. However, a protocol was designed to simplify the medication choice for the psychiatrist. This protocol was initially developed by two psychiatrists and two psychiatry residents on the research team using Canada’s guidelines (3) and a literature review throughout several meetings. Following this, the protocol was further revised by two more psychiatrists, a psychiatry resident, and a clinical psychologist. The pharmacotherapy protocol is summarized in Figure 1. All the psychiatrists involved in the care of patients were academically licensed psychiatrists in Canada familiar with Canadian Treatment Guidelines for Anxiety Disorders (3). During the intake appointment, the patient’s medication history (including any current medications) was collected. If a patient was taking medication for GAD that was not one of the recommended ones in the pharmacotherapy protocol (Figure 1; Sertraline, Escitalopram, Pregabalin, Duloxetine, Venlafaxine, Olanzapine, Risperidone, Benzodiazepines, Bupropion, Mitarzapine, Buspirone, Impipramine), they were switched to a suggested medication.

If a patient had never taken an SSRI or SNRI, they would commence the “primary medication” arm (Figure 1). The two classes of medications within the primary arm were described to the patient and, with the recommendation of the prescribing psychiatrist, the patient would begin either an SSRI (sertraline or escitalopram) or pregabalin/an SNRI (duloxetine or venlafaxine). If the patient had previously been deemed unresponsive to either an SSRI or an SNRI/pregabalin, they would commence the primary medication arm. The patient would start the medication class that they had not been previously deemed unresponsive to (e.g., if previously unresponsive to sertraline, the patient would commence the SNRI class). Previous unresponsiveness was defined as anxiety symptoms not improving after treatment with the maximum tolerated dose of the specific medication for 8 weeks. If a patient was deemed unresponsive to both an SSRI and an SNRI/pregabalin, they would commence the secondary medication arm. The two classes of medications within the second arm were described to the patient and, with the recommendation of the prescribing psychiatrist, they would begin either bupropion/mirtazapine or buspirone/imipramine.

At the patient’s second appointment (2 weeks on the medication), their medication was maintained and optimized, regardless of whether a response was reported. This optimization was an increase in medication within the recommended dosages if no response was evident, following the best practice guidelines (3). At the third appointment (4 weeks on the medication), the medication was optimized again if a partial response was reported or switched according to the “medication switch protocol” (Figure 1) if no response was reported. Partial response was defined as an improvement of 20% or greater in the GAD-7 score compared to the baseline. If the medication is switched (less than a 20% improvement in GAD-7 score compared to baseline), the six-week protocol would recommence with the new medication. At the fourth appointment (6 weeks on the medication), the dosage was optimized if the patient was responding well to the medication and reported an improvement greater than 50% if within the primary medication arm, or 20% if within the secondary medication arm, in GAD-7 score compared to baseline. If this were the case, the patient would remain on said medication for the 12-week study. If the patient did not present an improvement of more than 20% in their GAD-7 score compared to baseline, the medication would be switched according to the “medication switch protocol” (Figure 1) and the 6-week protocol would recommence. If the patient was in the primary medication arm and reported a 20–50% improvement in GAD-7 score compared to baseline after 6 weeks on the new medication, the medication was augmented with either olanzapine, risperidone or benzodiazepines (Figure 1).

#### 3.5.1 Medication switch

If a patient was unresponsive to medication after 4 or 6 weeks of administration (less than 20% improvement in GAD-7 score compared to baseline), their medication was switched to the other class (Figure 1). If the patient had a history of non-response to any of the four medication classes, these classes were removed as treatment options. If a patient started in the primary or secondary medication arm and had not previously demonstrated non-response to the second class of medications within that arm (less than 20% improvement in GAD-7), they were switched to the second class of pharmaceuticals in that arm. If a patient started in the primary arm and was previously unresponsive to the second class of pharmaceuticals within that arm, they began the secondary medication arm if necessary.

### 3.6 Ethics and data privacy

All procedures were approved by the Queen’s University HSREB. For privacy purposes, participants were only identifiable by an ID number on the platform and hard copies of the consent forms with participants’ identities were stored securely on-site and will be destroyed 5 years after study completion. Patient data was only accessible by the care providers directly assigned to that patient and only anonymized data was provided to the analysis team members. Participants could withdraw from the study at any point and request for their data to be removed from the analysis. However, since the collected data is considered a medical record, it cannot be permanently deleted for 10 years after treatment. OPTT is Health Insurance Portability and Accountability Act, Personal Information Protection and Electronic Documents Act, and Service Organization Control – 2 compliant. Additionally, all servers and databases are hosted in Amazon Web Service Canada cloud infrastructure which is managed by Medstack to ensure all provincial and federal privacy and security regulations are met. OPTT does not collect any identifiable personal information or IP addresses for privacy purposes. OPTT only collects anonymized metadata to improve its service quality and provide advanced analytics to the clinician team. OPTT encrypts all data, and no employee has direct access to patient data. All encrypted backups are kept in the S3 storage dedicated to Queen’s University, located in Kingston, Ontario, Canada.

### 3.7 Statistical analysis

Initially, all data were examined for missing, nonsensical, and outlying variables. Missing data from non-starters were treated as missing and not imputed at baseline analysis (i.e., analyzed on a per-protocol basis). The participant population of this study was intentionally over-sampled to account for drop-out/withdrawal. Data collection time points were at baseline, mid-point, and post-treatment. Using *t*-tests (paired sample for within group, two-sample for across group) and ANOVA (across groups) tests, demographic and completion statistics were analyzed for statistical differences between groups. Effect sizes were assessed between groups with Cohen’s d. Moreover, the difference in symptom changes between completers and non-completers was assessed. Using Mann–Whitney-*U* tests, demographic information was compared between patients who completed the program and those who withdrew prematurely to identify possible differences between the two. The sample size was determined using the GAD-7 score as the primary outcome, a 30% change is considered clinically significant (44–46). Therefore, a sample size of 55 participants in each arm of the study would be sufficient for detecting significant results with *p* = 0.05 and a power of 0.95.

## 4 Results

### 4.1 Participants

Participants (*N* = 115; e-CBT *N* = 41; Medication *N* = 41; Combination *N* = 33) were an average of 32.14 (SD = 12.12) years of age, with 63.48% of participants being female (*N* = 73). Across groups, there was no significant difference in age (*f* = 0.112, *p* = 0.885). There was a significant gender difference (*f* = 3.087, *p* = 0.050). Across groups, participants completed 7.51 (SD = 3.90) weeks, with 40.35% (*N* = 46) completing all 12 weeks. There was no significant difference between groups regarding the average number of weeks completed (*f* = 0.558, *p* = 0.574). Completion rates were calculated only including the sample of participants who completed more than 1 week, non-starters were not incorporated into these calculations. Please refer to Table 2 for demographic and completion statistics within each group.

**Table 2:** Demographic and completion statistics for each treatment arm.

	e-CBT (*N* = 41)	Medication (*N* = 41)	Combination (*N* = 33)
*Female (%)*	21 (51.22)	26 (63.42)	26 (78.79)
*Age (SD)*	32.85 (13.37)	31.95 (11.72)	31.49 (11.28)
*Complete (%)*	15 (36.59)	15 (36.59)	16 (48.49)
*Number of Weeks (SD)*	7.27 (3.83)	7.27 (3.89)	8.12 (4.05)

**Figure 2:**
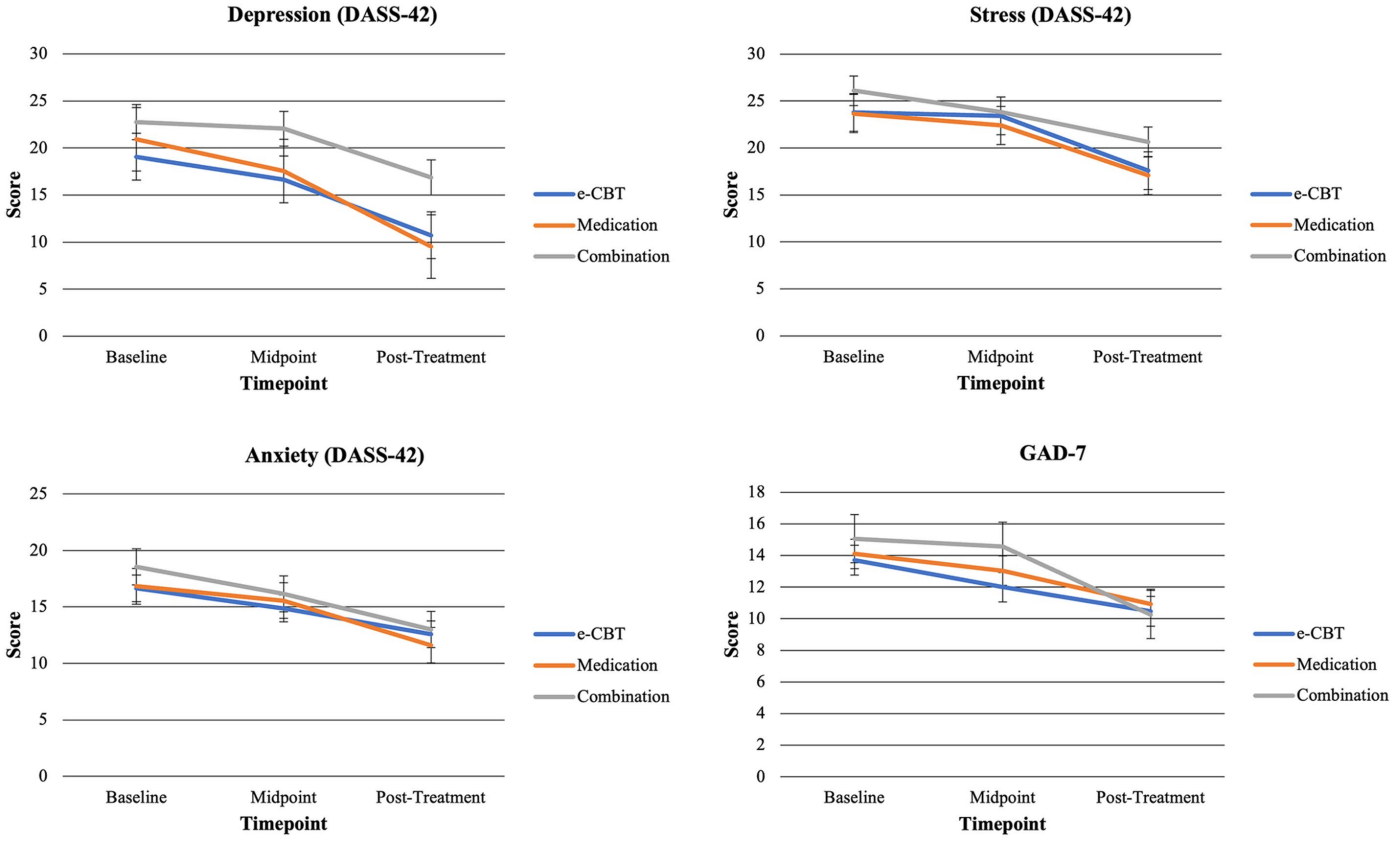
Symptom severity questionnaire score means across arms and time points. DASS-42 is broken into three subscales (Depression, Anxiety, Stress) for all analyses.

**Figure 3:**
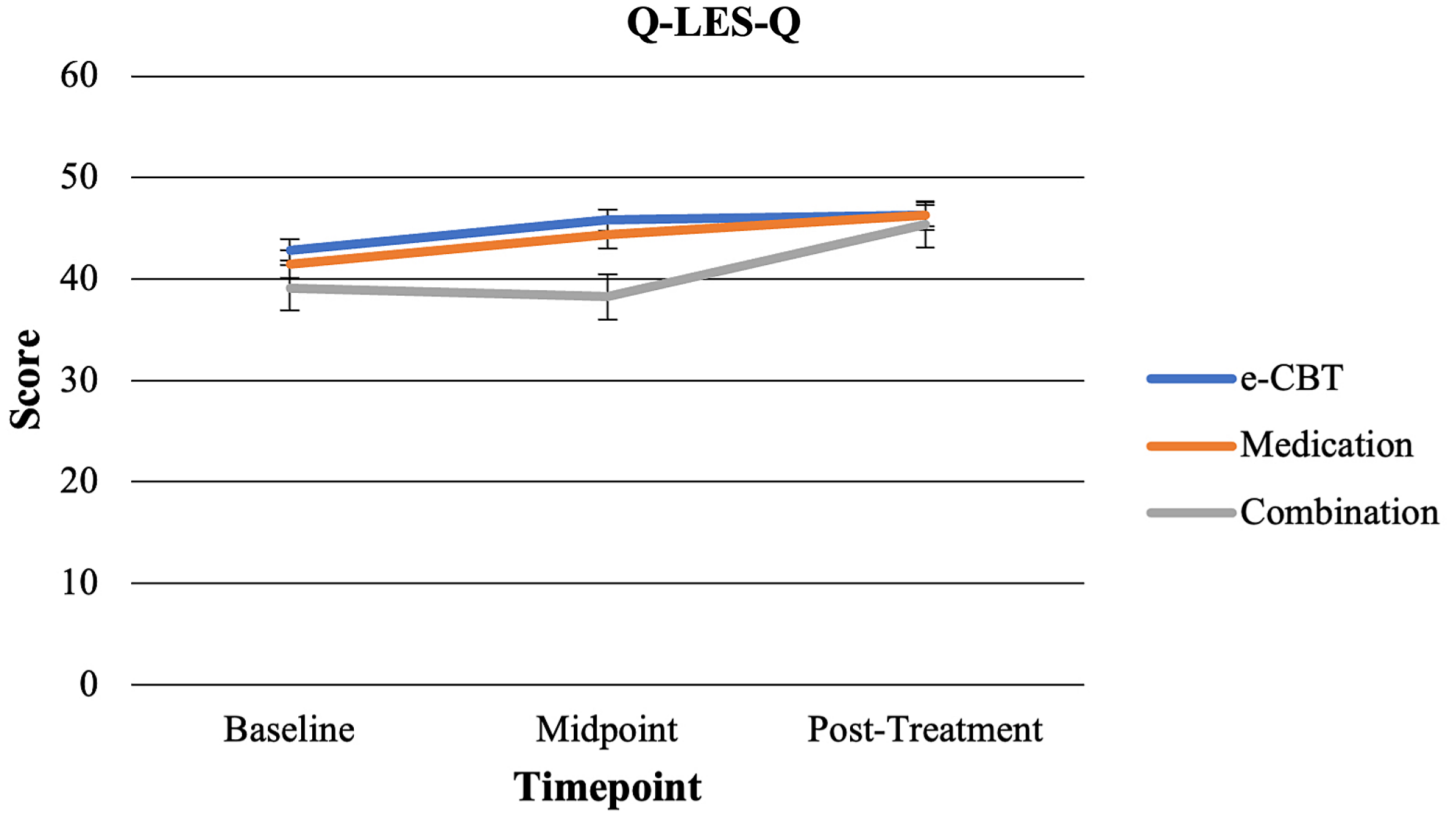
Changes in quality of life across time points for all three arms.

At baseline, there were no significant differences between groups regarding DASS-42 subscales (depression *f* = 0.924, *p* = 0.400; anxiety *f* = 0.480, *p* = 0.620; stress *f* = 0.949, *p* = 0.390). Additionally, there were no significant differences found at baseline across groups for GAD-7 (*f* = 0.763, *p* = 0.469) or Q-LES-Q (*f* = 1.701, *p* = 0.187) scores. Figure 2 shows symptomatology questionnaire scores at collection time points between groups. Figure 3 shows changes in quality of life (Q-LES-Q). Mann–Whitney *U* Tests revealed that group (*Z* = −0.977, *p* = 0.329) and gender (*Z* = −1.253, *p* = 0.210) did not have significant effects on the completion status. However, the test did reveal that age had a significant effect on completion status (*Z* = −3.094, *p* = 0.002, Mann–Whitney *U* = 1045.500). The average age of completers was 36.70 (SD = 13.64) years, and non-completers were 29.10 (SD = 9.99) years. Females had a completion percentage of 43.84% (*N* = 32), and males had a completion percentage of 33.33% (*N* = 14).

### 4.2 Within-group treatment effects

Tables 3–5 show within-group paired t-test results across all time points and questionnaires for the e-CBT, Medication, and Combination Treatment Arms, respectively.

**Table 3:** Paired *t*-test results within the e-CBT group across all time points for questionnaire score changes.

	*t*	df	One-sided *p*	Two-sided *p*
Depression				
*T0 → T1*	−0.328	22	0.373	0.746
*T1 → T2*	1.908	13	0.039	0.079
*T0 → T2*	1.551	14	0.072	0.143
Anxiety				
*T0 → T1*	0.619	22	0.271	0.542
*T1 → T2*	1.392	12	0.095	0.189
*T0 → T2*	1.259	13	0.115	0.230
Stress				
*T0 → T1*	−0.040	22	0.484	0.968
*T1 → T2*	2.037	13	0.031	0.063
*T0 → T2*	1.049	14	0.156	0.312
GAD-7				
*T0 → T1*	1.803	23	0.042	0.085
*T1 → T2*	0.849	13	0.206	0.411
*T0 → T2*	2.442	14	0.014	0.028
Q-LES-Q				
*T0 → T1*	−1.886	23	0.036	0.072
*T1 → T2*	−0.282	13	0.391	0.783
*T0 → T2*	−1.547	14	0.072	0.144

An increase in Q-LES-Q is considered an improvement, whereas other questionnaires calculate an improvement in a decrease in score.

**Table 4:** Paired *t*-test results within the medication group across all time points for questionnaire score changes.

	*t*	df	One-sided *p*	Two-sided *p*
Depression				
*T0 → T1*	0.159	24	0.437	0.875
*T1 → T2*	3.236	14	0.003	0.006
*T0 → T2*	2.522	14	0.012	0.024
Anxiety				
*T0 → T1*	0.737	24	0.234	0.468
*T1 → T2*	2.053	14	0.030	0.059
*T0 → T2*	2.255	14	0.020	0.041
Stress				
*T0 → T1*	0.645	24	0.262	0.525
*T1 → T2*	2.281	14	0.019	0.039
*T0 → T2*	1.380	14	0.095	0.189
GAD-7				
*T0 → T1*	1.568	25	0.065	0.129
*T1 → T2*	0.774	14	0.226	0.452
*T0 → T2*	2.990	14	0.005	0.010
Q-LES-Q				
*T0 → T1*	−2.009	25	0.028	0.055
*T1 → T2*	−0.951	14	0.179	0.358
*T0 → T2*	−1.547	14	0.072	0.144

An increase in Q-LES-Q is considered an improvement, whereas other questionnaires calculate an improvement in a decrease in score.

**Table 5:** Paired *t*-test results within a combination group across all time points for questionnaire score changes.

	*t*	df	One-sided *p*	Two-sided *p*
Depression				
*T0 → T1*	1.305	19	0.104	0.207
*T1 → T2*	1.996	13	0.034	0.067
*T0 → T2*	2.911	13	0.006	0.012
Anxiety				
*T0 → T1*	1.809	19	0.043	0.086
*T1 → T2*	1.897	13	0.040	0.080
*T0 → T2*	2.164	13	0.025	0.050
Stress				
*T0 → T1*	1.409	19	0.087	0.175
*T1 → T2*	1.617	13	0.065	0.130
*T0 → T2*	2.537	13	0.012	0.025
GAD-7				
*T0 → T1*	1.041	20	0.155	0.310
*T1 → T2*	2.809	14	0.007	0.014
*T0 → T2*	2.731	14	0.008	0.016
Q-LES-Q				
*T0 → T1*	−0.592	19	0.280	0.561
*T1 → T2*	−3.662	13	0.001	0.003
*T0 → T2*	−3.401	13	0.002	0.005

An increase in Q-LES-Q is considered an improvement, whereas other questionnaires calculate an improvement in a decrease in score.

### 4.3 Between-group treatment effects

*Mid-Point*: At the midpoint, no significant differences were found between groups regarding DASS-42 scores (Depression *f* = 1.175, *p* = 0.315; Anxiety *f* = 0.095, *p* = 0.909; Stress *f* = 0.103, *p* = 0.902). Additionally, no significant differences in GAD-7 (*f* = 1.259, *p* = 0.291). However, the Q-LES-Q score at the midpoint for the Combination Treatment Arm was significantly lower than the e-CBT (*t* = 2.506, *p* = 0.008) and Medication (*t* = 2.169, *p* = 0.018) Treatment Arms.

*Post-Treatment*: At post-treatment, there was no significant difference in any questionnaire scores (Depression *f* = 2.284, *p* = 0.115; Anxiety *f* = 0.077, *p* = 0.926; Stress *f* = 0.377, *p* = 0.688; GAD-7 *f* = 0.052, *f* = 0.949; Q-LES-Q *f* = 0.048, *p* = 0.953). There was also no significant difference in change in score across groups (Table 6) for any questionnaire (Depression *f* = 0.622, *p* = 0.542; Anxiety *f* = 0.081, *p* = 0.923; Stress *f* = 0.703, *p* = 0.501; GAD-7 *f* = 0.149, *p* = 0.862; Q-LES-Q *f* = 0.084; *p* = 0.920). A complete breakdown of scores across timepoints is provided in Table 7.

**Table 6:** The average change in questionnaire score, with standard deviation and percent change.

	e-CBT (SD)	Medication (SD)	Combination (SD)
*Depression*	−3.07 (7.66), 43.73%	−5.33 (8.19), 54.44%	−6.07 (7.16), 25.93%
*Anxiety*	−3.40 (8.32), 24.42%	−3.80 (6.53), 31.07%	−4.47 (7.09), 29.99%
*Stress*	−3.00 (11.07), 25.99%	−3.47 (9.73), 27.86%	−7.00 (9.43), 20.88%
*GAD-7*	−3.67 (5.82), 23.64%	−3.40 (4.41), 22.45%	−4.47 (6.33), 31.83%
*Q-LES-Q*	+3.00 (7.51), 7.90%	+3.00 (7.51), 11.52%	+4.27 (13.2), 15.94%

An increase in Q-LES-Q is considered an improvement, and a decrease in all other questionnaire cores is considered an improvement.

**Table 7:** Questionnaire score means and standard deviations for each group across timepoints.

	T0 (SD)	T1 (SD)	T2 (SD)
e-CBT (T0 *N* = 41; T1 *N* = 23; T2 *N* = 15)			
*Depression*	19.07 (11.41)	16.65 (13.55)	10.73 (9.16)
*Anxiety*	16.63 (9.57)	14.87 (10.24)	12.57 (9.17)
*Stress*	23.78 (8.65)	23.44 (11.89)	17.60 (12.16)
*GAD-7*	13.71 (5.02)	12.00 (6.19)	10.47 (5.40)
*Q-LES-Q*	42.88 (9.38)	45.83 (11.74)	46.27 (8.84)
Medication (T0 *N* = 41; T1 *N* = 25; T2 *N* = 15)			
*Depression*	20.97 (12.30)	17.56 (11.44)	9.53 (9.05)
*Anxiety*	16.83 (8.69)	15.56 (9.11)	11.60 (8.68)
*Stress*	23.66 (8.58)	22.40 (11.05)	17.07 (11.54)
*GAD-7*	14.10 (4.75)	13.04 (5.00)	10.93 (5.06)
*Q-LES-Q*	41.49 (9.11)	44.39 (10.86)	46.27 (8.84)
Combination (T0 *N* = 33; T1 *N* = 20; T2 *N* = 16)			
*Depression*	22.76 (10.96)	22.05 (11.61)	16.86 (11.27)
*Anxiety*	18.55 (8.74)	16.15 (9.57)	13.00 (11.66)
*Stress*	26.09 (7.68)	23.85 (10.35)	20.64 (11.98)
*GAD-7*	15.06 (4.46)	14.57 (5.04)	10.27 (6.76)
*Q-LES-Q*	39.12 (7.35)	38.25 (7.36)	45.36 (9.58)

*Completers vs. Non-Completers*: Across groups, completers had significantly lower baseline depression (*t* = 2.956, *p* = 0.002; completer mean = 17.00, SD = 10.88; dropped out mean = 23.32, SD = 11.46) and stress (*t* = 2.066, *p* = 0.021; completer mean = 22.46, SD = 9.54; dropped out mean = 25.70, SD = 7.24) scores compared to those that dropped out. Within the e-CBT group, completers had significantly lower baseline depression (*t* = 2.374, *p* = 0.011; completer mean = 13.80, SD = 10.64; dropped out mean = 22.12, SD = 10.90) and stress (*t* = 1.842, *p* = 0.037; completer mean = 20.60, SD = 10.47; dropped out mean = 25.62, SD = 6.97) scores compared to those that dropped out. In the medication group, completers had significantly lower baseline depression (*t* = 2.556, *p* = 0.007; completer mean = 14.87, SD = 9.40; dropped out mean = 24.42, SD = 12.59) and stress (*t* = 1.822, *p* = 0.038; completer mean = 20.53, SD = 9.93; dropped out mean = 25.46, SD = 7.31) scores compared to those that dropped out. There were no significant differences in baseline scores between completers and those that dropped out in the combination group.

## 5 Discussion

This study compared the treatment efficacy of an online psychotherapy program to medication, and a combination of the two in patients with GAD. While e-CBT has been established as a quality intervention for treating anxiety compared to control and in-person CBT (47), there is limited literature comparing it to medications. Moreso, while current clinical guidelines do not support a routine combination of CBT and medication for treating GAD^3^, there is limited knowledge on whether this also applies to e-CBT with medication.

This study adds to the evidence that e-CBT is a viable option for GAD, with patients in the e-CBT arm seeing significant improvements in GAD-7 scores. There were no significant differences in questionnaire scores or changes in questionnaire scores post-treatment, suggesting that all three treatment options offer comparable results. Although there were no significant differences across groups in questionnaire scores, the within-group analysis revealed the medication arm had significant improvements in depression, anxiety, and GAD-7 scores and the combination arm had significant improvements in all five questionnaires. This suggests that similar to current guidelines for in-person CBT, routine augmentation of e-CBT with medication is not supported, it may be beneficial to some patients. Moreover, there was no significant difference in completion percentage, or the average number of weeks completed across the three treatment options.

Comparing patients who completed the 12 weeks to those that did not across all groups, completers presented with significantly lower depression and stress scores on the DASS-42 at baseline. The same was true for within-group comparison in the e-CBT and medication arms, but not the combination arm. More work should investigate whether these sections of the DASS-42 could be used as possible treatment attrition indicators. While younger patients were more likely not to complete the study, it should be noted that the average ages of both groups were adults (completers = ~37 years; non-completers = 29 years).

### 5.1 Limitations

All three arms in this study had somewhat low attrition rates. However, this is not uncommon for psychiatric intervention trials, with several meta-analyses finding pooled dropout rates as high as 60%, with online psychotherapeutic interventions having high dropout rates (48–52). This is often due to the nature of the psychiatric population. Patients may feel the treatment is ineffective early in the trial, may need additional support, or may have concerns about the safety or security of the intervention. In this study, it was found that higher depression and stress scores at baseline were also associated with a higher likeliness of dropout, indicating that more severe symptoms may require more support to ensure a patient completes the program. Comorbidities, symptom severity, and expectations of treatment all contribute to treatment adherence in patients with anxiety (53). While there was a high dropout, however, there was no significant difference in completion percentage or weeks completed between the three interventions. This suggests that attrition rates were independent of treatment type. More work should be done in the future to investigate ways to improve the attrition rates, perhaps incorporating forms of augmented care with differing levels of care provider engagement to find the optimal combination to retain patients. Moreover, participants were removed if they had more than two consecutive weeks of missing e-CBT sessions without contact. This could have created a bias toward patients with the time, resources, or motivation for treatment being included in analysis. Additionally, this study employed a treatment allocation design that allowed patients to select which arm they were enrolled in. While randomization would have provided balance to possible unknown confounding variables, a treatment-preference design mimics a clinical setting. This study aimed to understand whether e-CBT is a quality treatment option compared to medication or a combination. With previous research already demonstrating that e-CBT is similarly beneficial compared to in-person psychotherapies, having a treatment-preference model allows for the findings to be applied more closely to a real-world setting (54–57). Moreso, given that the medications prescribed were first-line, tolerable medications that followed guidelines, there was not an elevated risk of allowing a patient to make an informed decision on their treatment. It has also been shown that empowering a patient to choose their intervention can improve treatment outcomes (24–27). Finally, given the relatively short timeline of this program (i.e., 12 weeks), medications may not have demonstrated their full efficacy in the period. Future work should incorporate a longer time-frame with additional follow-up assessments to observe these effects.

### 5.2 Conclusion

This study found that e-CBT, medication, and a combination of the two all resulted in symptom improvement in patients with GAD. Moreover, there was no significant difference in the efficacy of any treatment. Results suggest that while a routine combination of the two is not necessary, it could be beneficial to some patients. Given that each form of treatment has its own set of benefits and drawbacks and that there is no difference in treatment efficacy, allowing the patient to make an informed decision on which intervention fits their lifestyle and personal views is recommended.

## Data availability statement

The raw data supporting the conclusions of this article will be made available by the authors, without undue reservation.

## Ethics statement

The studies involving humans were approved by Queen’s University Health Sciences and Affiliated Teaching Hospitals Research Ethics Board. The studies were conducted in accordance with the local legislation and institutional requirements. The participants provided their written informed consent to participate in this study.

## Author contributions

All authors listed have made a substantial, direct, and intellectual contribution to the work and approved it for publication.
